# AgSCN as a new hole transporting material for inverted perovskite solar cells

**DOI:** 10.1038/s41598-023-35081-z

**Published:** 2023-05-16

**Authors:** Ahmed Mourtada Elseman

**Affiliations:** Electronic and Magnetic Materials Department, Central Metallurgical Research and Development Institute (CMRDI), Helwan, P.O. Box 87, Cairo, 11421 Egypt

**Keywords:** Materials science, Materials for devices, Materials for optics, Structural materials, Energy

## Abstract

A novel HTM based on silver thiocyanate (AgSCN) was designed to be useable in p-i-n perovskite solar cells (PSCs). With mass yield, the AgSCN was synthesized in the lab and elucidated by XRD, XPS, Raman spectroscopy, UPS, and TGA. The production of thin, highly conformal AgSCN films that allow for quick carrier extraction and the collection was made possible by a fast solvent removal approach. Photoluminescence experiments have shown that adding AgSCN has improved the ability to transfer charges between HTL and perovskite layer compared to PEDOT:PSS at the interface. Crystallographic discrepancies in the polycrystalline perovskite film are discovered upon further examination of the film's microstructure and morphology, pointing to the development of templated perovskite on the surface of AgSCN. In comparison to devices due to the well-known PEDOT:PSS, the open circuit voltage (V_OC_) is increased by AgSCN with its high work function by 0.1–1.14 V (1.04 V for PEDOT:PSS). With a power conversion efficiency (PCE) of 16.66%, a high-performance PSCs are effectively generated using CH_3_NH_3_PbI_3_ perovskite compared to 15.11% for controlled PEDOT:PSS devices**.** The solution-processed inorganic HTL was demonstrated employing straightforward in order to build durable and effective flexible p-i-n PSCs modules or their use as a front cell in hybrid tandem solar cells.

## Introduction

Research on perovskite solar cells (PSCs) has come a long way in the previous decade. PSC has achieved a high degree of power conversion efficiency (PCE), over 25.7%; nonetheless, various issues, including low stability and high cost, continue to prevent its practical deployment^1–4^. A traditional standard PSC is composed of a conducting TCO substrate (ITO or FTO), an electron transporting/extraction layer (ETL), an absorber layer from perovskite, a hole transporting/extraction layer (HTL), and back contact as electrode^5,6^. Because of their successful hole transporting/extraction capacity and interface adjustment that hinder electron transfer from the absorber layer (perovskite) to metallic anodes (hole transportation materials (HTMs) or HTLs) are important for efficacious PSCs^7^. Advanced HTMs like PTAA, Spiro-MeOTAD and PEDOT: PSS are available for commercialization today^8–10^. However, limited crystallinity, poor mobility, high cost, and potential air deterioration due to humidity are only some issues with these organic HTMs^11,12^. Inorganic, thermally, and chemically compatible alternatives at low treatment temperatures and very stable are extremely rare^13,14^. The creation of updated, low-cost, convenient-to-get HTM alternatives for highly effective PSCs is, of course, imperative. Appropriate HTMs require high mobility, the highest degree of occupied molecular orbital (HOMO) energy, and stable chemical/physical properties^14,15^. Considering their high mobility, stability, ease of synthesis, and low cost, inorganic p-type semiconductors are a better option than organic HTMs^13,14,16^.

Organic hole conductor of PEDOT:PSS^17^ is replaced by inorganic HTM p-type material based on inverted planar PSCs. Because the V_OC_ of a planar heterojunction PSC is significantly established via the perovskites/charge-transporting interlayers interfaces, the possible energy loss at the interface between PEDOT:PSS and CH_3_NH_3_PbI_3_ that results in reduced V_OC_^18^. For example, when compared to PEDOT:PSS, CuSCN is distinguished with energy levels of VB =  − 5.3 eV and CB =  − 1.8 eV which is consistent with CH_3_NH_3_PbI_3_ (VB =  − 5.4 eV). Moreover, CuSCN delivers better transparency throughout the UV–Vis–NIR range with a wide band-gap (E_g_) of 3.6 eV, making it easier for photoactive materials to absorb more light in an inverted structure to generate higher photocurrent^19^. This study employs AgSCN as a substitute inorganic HTM for CuSCN^20^. Notably, other than their use as a Cu and Ag doping source in a complementary metal dichalcogenide (CdTe) solar cell^21^, solution-processed CuSCN, and AgSCN have not been reported as inorganic HTL-based PSCs. CuSCN's advantages lie in the fact that it can serve as both a hole transport layer and a source of Cu doping, while AgSCN, with its greater resistivity, may serve just as a source of Ag doping with a slower diffusion rate^21^. In Table S1 (SI), we summarized the photovoltaic parameters of inverted PSCs based on CuSCN as inorganic HTM during the period of 2015–2020 in order to stand up for efficiency compared to the new results of AgSCN. It is known that CuSCN consists of Cu^+^, which is less stable than Cu^2+^ and undesirable for chemical stability. The structure relies on the enthalpy of ions as they bind to other molecules (hydration energy). The Cu^2+^ ion has a higher charge density than Cu^+^ ion, creating much stronger bonds that release extra energy^22–24^.

In this work, AgSCN as HTL is investigated through the solution processing of inverted PSC architecture based on CH_3_NH_3_PbI_3_ as an absorber layer for the first time. The p-i-n type device architecture consists of ITO/AgSCN/CH_3_NH_3_PbI_3_/PCBM/BCP/Ag. A maximum PCE of 16.66% was obtained in preliminary experiments by employing AgSCN-HTL.

## Methods

### Materials

#### Synthesis of silver thiocyanate (AgSCN) as HTM

**Figure 1:**
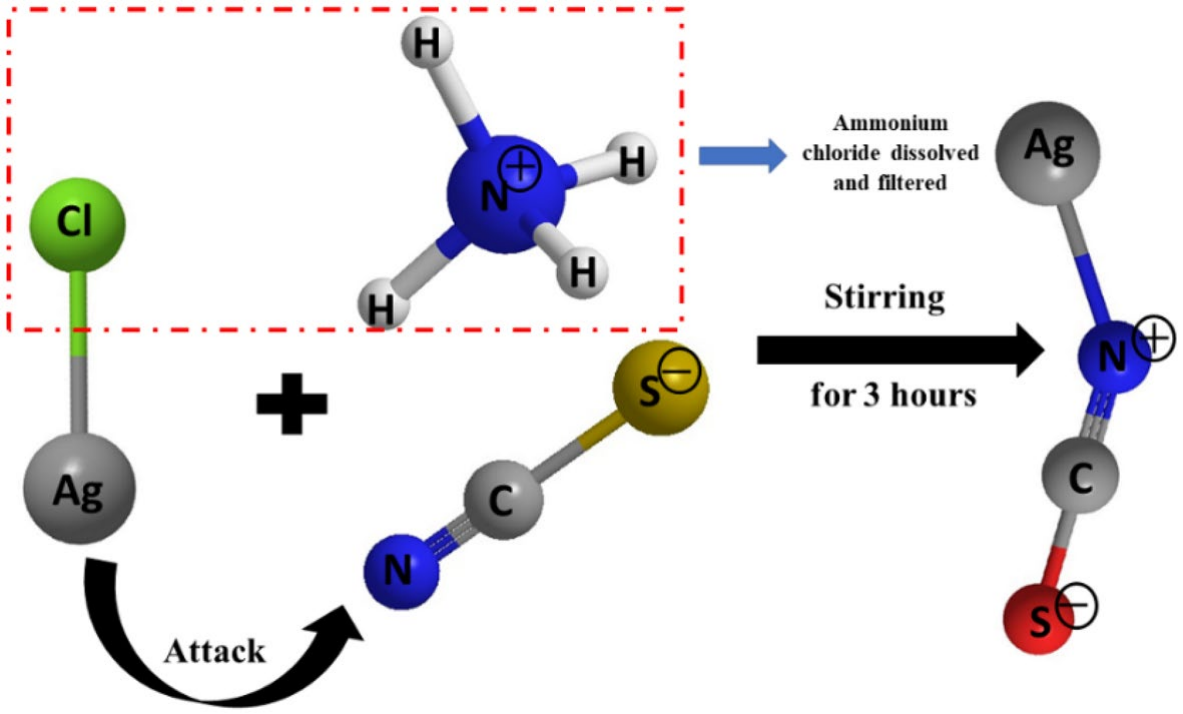
Molecular structures and synthetic procedures for AgSCN.

0.1 mol/L of silver chloride (AgCl) powder was dissolved in double-distilled (DD) water for 1 h under continuous stirring. After finishing the stirring, the clear silver chloride solution was stored at room temperature between 0 and 5 °C for 1 day. 0.1 mol/L of ammonium thiocyanate (NH_4_SCN) was dissolved in DD water under continuous stirring for 1 h. Then, a solution from silver chloride was loaded in a conical flask (300 mL) and a solution from NH_4_SCN was added gradually with stirring for 3 h. A precipitate from AgSCN nanostructures was precipitated out. The obtained precipitate was washed many times with water before being dehydrated at 60 °C for 6 h in a vacuum oven. The reaction mechanism of one step is as the following Fig. 1.

### Device fabrication

An ultrasonic cleaning system was used to clean the ITO substrate, the size of the ITO-coated glass (2 cm × 2 cm), which was initially submerged in a cleansing agent combination and deionized water before being rinsed many times with deionized water. Spinning for AgSCN in dipropyl sulfide with different concentrations was accustomed to HTL preparation at 3000 rpm on ITO substratum for 30 s at room temperature. The deposition of AgSCN thin film is done in a dry glove box with relative humidity (RH) between 25 and 30 and then left for at least 30 min before the deposition of the perovskite layer starts. The PEDOT:PSS, as reference HTL, was spin-coated in the atmosphere (out of the glove box) at 6000 rpm for 40 s, according to the literature of^18^. Afterward, the HTL-coated substrate was transferred to the glove box for the next step. The active layer on HTL was deposited by spin coating a perovskite precursor solution at 4000 rpm for 30 s. After 12 s, the 350 $\mu$ L of chlorobenzene was cast vertically. After 90 s of annealing at 50 °C, the spin-coated film was transferred to a different hotplate and annealed at 100 °C for 10 min. The solution of PC_61_BM was deposited at 4000 rpm for 30 s for the ETL on the perovskite absorber sheet. For 30 s, the buffer layer of the BCP solution was spun at 2000 rpm to cover the surface. Then, the cells were transferred to the chamber of the thermal evaporator to heat evaporation of Ag at 120 nm, eventually formed the back contact. The overlap between the electrode ITO and the Ag reveals an active area of 0.1 cm^2^.

### Characterization

In SHIMADZU, Japan, an XRD-7000 X- diffractometer was used to determine the XRD pattern. We used TGA/DTA to evaluate a STA504. To measure UV- absorption spectra, a Shimadzu UV-2550 spectrometer was employed. The perovskite layer's grain size was measured using field-scanning electron microscopy (SEM, JSM-6700F). Using a Thermo Fisher ESCALAB 250Xi, we analyzed the ultraviolet photoelectrons' spectra. The AgSCN and PEDOT:PSS samples were characterized by Hall effect measurements in Van der Pauw geometry (Ecopia HMS-3000), with the four soldering points placed in the corners of substrates. A 660D electrochemical workstation (Shanghai Chen hua Instrument Co., Ltd., China) was employed to determine the AC impedance spectrum. The Newport simulator (model 94043A) was used to acquire the J-V curves, while the simulated light intensity was set to 100 mW/cm^2^ and measured using a Keithley 2400 source meter. With a ten mV voltage step and zero milliseconds of delay, we implemented the − 1.2 to 0.2 V backward scan, and the measured condition was 25–30 °C. A lock-in amplifier (SR-830) was used to get the photocurrent generated by the monochromatic light that was manipulated. Device EQE was determined by calculating the photocurrent measured and the light intensity.

## Results

**Figure 2:**
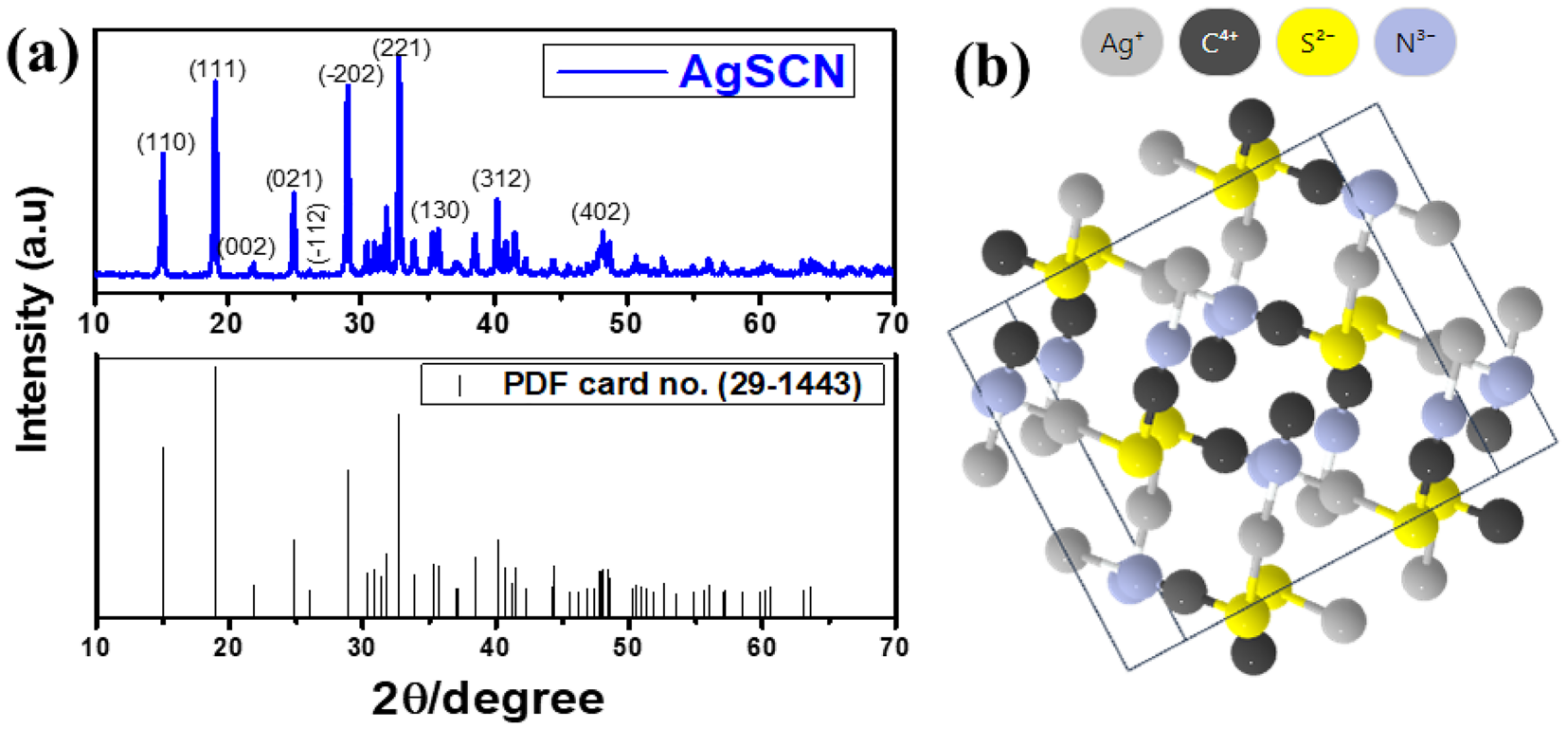
(**a**) X-ray diffraction analysis and corresponding JCPDS card of synthesized HTM of AgSCN. (**b**) Molecular geometry of AgSCN crystal.

As seen in Fig. 1, the new HTM was synthesized using traditional precipitation reactions in reasonable yields. The features of the photophysical, electrochemical, thermal, and electronic structures and the properties related to the HTM were systematically investigated. See Fig. 2a for X-ray diffraction study of AgSCN particles. The XRD spectrum peaks are clearly different. All can be precisely indexed to crystalline AgSCN, not only in peak positions but also in relative strength. All patterns matched with AgSCN very well and agreed with JCPDS: 29–1443. In-group space: C2/c, *a* = 8.774 Å, *b* = 7.972 Å, and *c* = 8.182 Å also matched. The indexed peaks at 2θ = 15.03°, 18.97°, 21.77°, 24.87°, 25.99°, 28.88°, 33.82°, 36.96°, 40.20°, 48.42° were allocated to the mono-clinical planes of AgSCN crystal (110), (111), (002), (− 112), (−202), (221), (130), (312), (402)^25,26^. Figure 2b demonstrates that AgSCN is monoclinic in the cell, with eight molecules. The SCN^−^ groups, with a bond angle of 179.6(5)°, have an almost linear molecular geometry. The interaction length of low Ag–Ag ranges from 0.3249(2) to 0.3338(2) nm, like a zigzag in one dimension sequence in AgSCN^27^. As evidenced by the sharp shapes of the prominent peaks, there was a scarcity of defects in AgSCN, which improve photoinduced charges by recombination centers. Thus, AgSCN can possibly be used as HTL with high hole extraction capability. On the other hand, for characterizing the AgSCN, the Raman spectroscopy analysis was further carried out. Figure S1, supplementary information (SI) indicates the symmetrical Vibrations that prolong of SCN and CN in the AgSCN, resulting in peaks of 2150 and 710 cm^−1^^28^.

**Figure 3:**
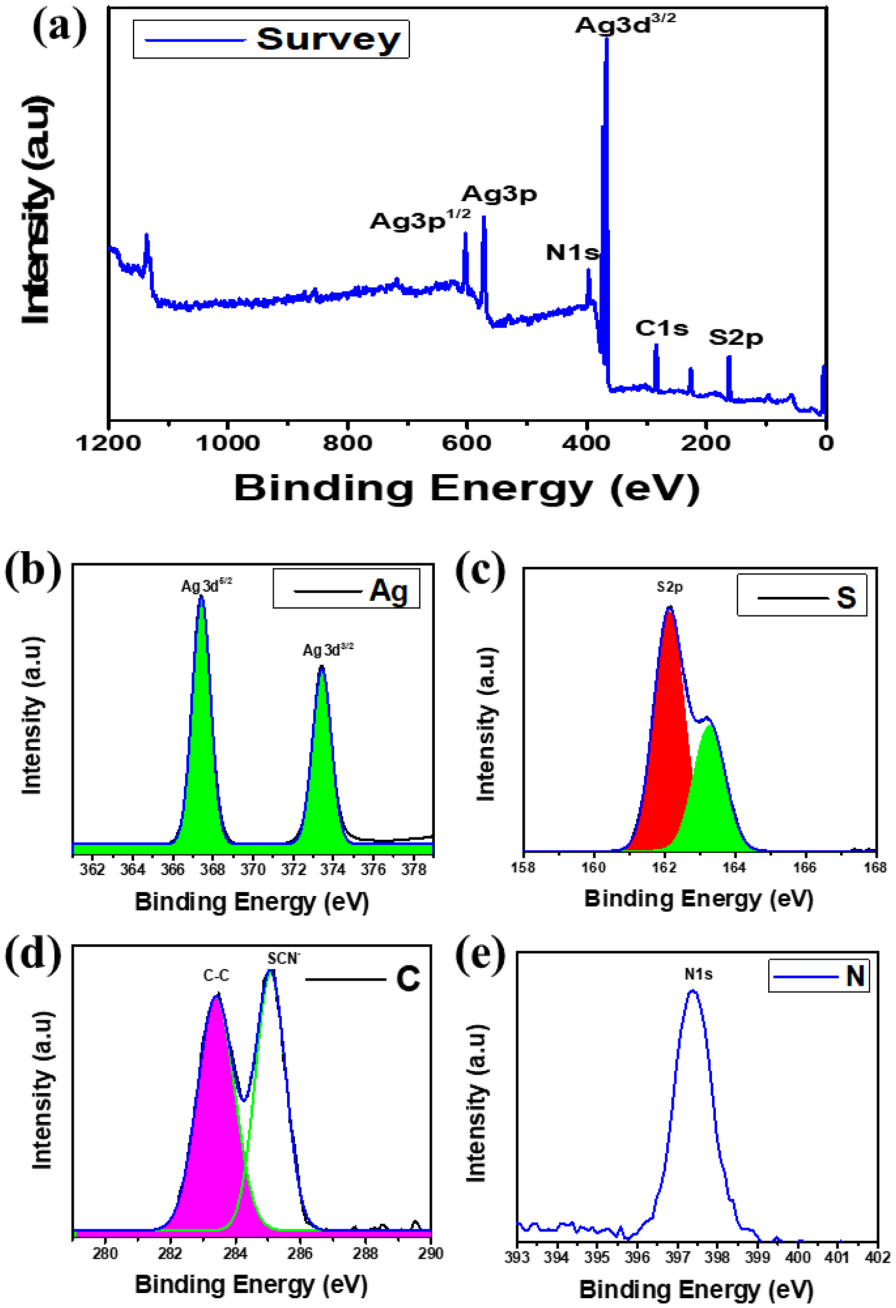
X-ray photoelectron spectroscopy analysis includes (**a**) the survey XPS of the product indicating all the peaks of the present invented AgSCN, (**b**) Ag 3d, (**c**) S 2p, (**d**) C 1s, and (**e**) N 1s.

An overview of the AgSCN's XPS is shown in Fig. 3a, showing all AgSCN peaks^29^. Two peaks are observed in Fig. 3b, indicating the Ag 3d XPS spectra. Ag 3d_5/2_ at 368.4 eV and Ag 3d_3/2_ at 374.4 eV, respectively, suggesting the monovalent of Ag condition. In Fig. 3c, the peaks at 163.2 and 164.4 eV could be assigned to S 2p of SCN^−^. The peaks in Fig. 3d at 284.5 and 286.25 eV correspond to groups C–C and SCN − . In Fig. 3e, the N 1 s XPS spectrum shows that the peak that appeared at 398.3 eV belongs to the category C=N–C^30^.

**Figure 4:**
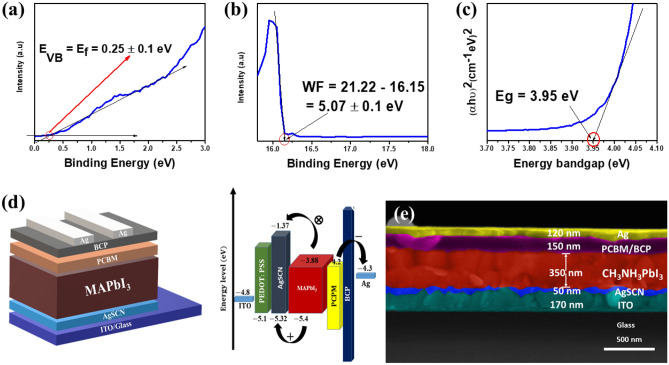
(**a**) Ultraviolet photoelectron spectroscopy (UPS) describes the energy of valence band maximum (VBM), and (**b**) work function (WF) of AgSCN sample. (**c**) Direct bandgap transition by Tuac’s calculation of AgSCN. (**d**) Device architecture of AgSCN based (p-i-n) PSC with a schematic energy band diagram positioned in relation to a vacuum. (**e**) Cross-section SEM image of PSC on the substrate with AgSCN as HTL.

A suitable alignment of energy levels between perovskite ad HTM is important for suppressing the energy losses in PSCs. In Fig. 4 and Fig. S2 (SI), the UPS spectra shown here have been used to investigate the work function of AgSCN thin film. The total wide-range UPS is presented in Fig. S2 (SI). As revealed in Fig. 4a,b, the Fermi energy level of AgSCN was significantly downshifted relative to the nearest structure of CuSCN, as reported^31–33^. For AgSCN, for the second cutoff, the work function is all around 5.07 eV which is calculated from Φ_AgSCN_ = 16.15–21.22 eV = –5.07 ± 0.1 eV^13,34–36^; for more details about the calculation is found in the SI. By collecting the Fermi level and work function value, the valence band (VB) will equal – 5.32 ± 0.2 eV. The increased energy level of AgSCN may greatly aid band alignment between AgSCN and perovskites. The AgSCN, therefore, has a marginally higher VB of – 5.32 eV compared to PEDOT:PSS (as experimentally measured in our previous work^37^) and CuSCN with a – 5.1 and − 5.3 eV, respectively^18,38^. AgSCN's work function was a better fit for hole transport and electron blocking than that of any other perovskite studied (– 5.4 eV). The enhanced work function may also boost the V_OC_ of the device by increasing the possible distinction between the HTL VB and the perovskite (VB)^18^. In order to determine the CB of AgSCN, we obtained the optical band for AgSCN thin-film by *αhʋ* = *A(hy − E*_*g*_*)*^*n*^ (Tauc's equations) through transmittance spectra^39^ as shown in Fig. 4c. The linear curve specifies that the optical bandgap (E_g_) of AgSCN is equal to 3.95 ± 0.1 eV. Hence, the CB minimum is up to − 1.37 ± 0.1 eV. As shown in the schematic of energy level in Fig. 4d, the favorable energy match between AgSCN and perovskite layer is noticed compared to PEDOT:PSS, which is used as a famous HTL in inverted structure PSCs. ITO/PEDOT:PSS and ITO/AgSCN thin films’s transmission spectra are shown in Fig. S3. Compared to ITO/PEDOT:PSS, the AgSCN's transmission has no change with the bit of more significant transmission, which has no impact on the conversion output.

The configuration of the PSC devices consisting of ITO/AgSCN/MAPbI_3_/PCBM/BCP/Ag revealed in Fig. 4d has been selected to learn how our HTL layer affects the functionality of perovskite devices. The experimental section includes a complete description of how to make the devices. A Field emission scanning electron microscopy (FE-SEM) picture of the cross-section of the inverted device design is shown in Fig. 4e of glass/ITO, AgSCN HTL with CH_3_NH_3_PbI_3_, spin-coated PCBM as ETL, BCP as a buffer layer, and finally Ag as top electrode. The improved and efficient cell based on AgSCN HTL is shown in SEM image to have a homogeneous deposition along its length. The device fabrication process started with an extremely thin layer of AgSCN (50 nm) on top of the ITO glass. Next, a homogeneous and dense 350 nm thick MAPbI_3_ film was deposited on the AgSCN substrate or PEDOT:PSS as a reference with a high-quality perovskite layer and excellent crystallinity, both of which are expected to aid in the efficient production and transfer of charge. After that, a layer of PCBM/BCP with a thickness of around 150 nm was applied on top. Back contact was ultimately provided via 120 nm based on Ag electrode. The schematic Fig. 4 level diagram, which is based on the aforementioned data, elucidates the efficient transmission and extraction of electrons and holes.

**Figure 5:**
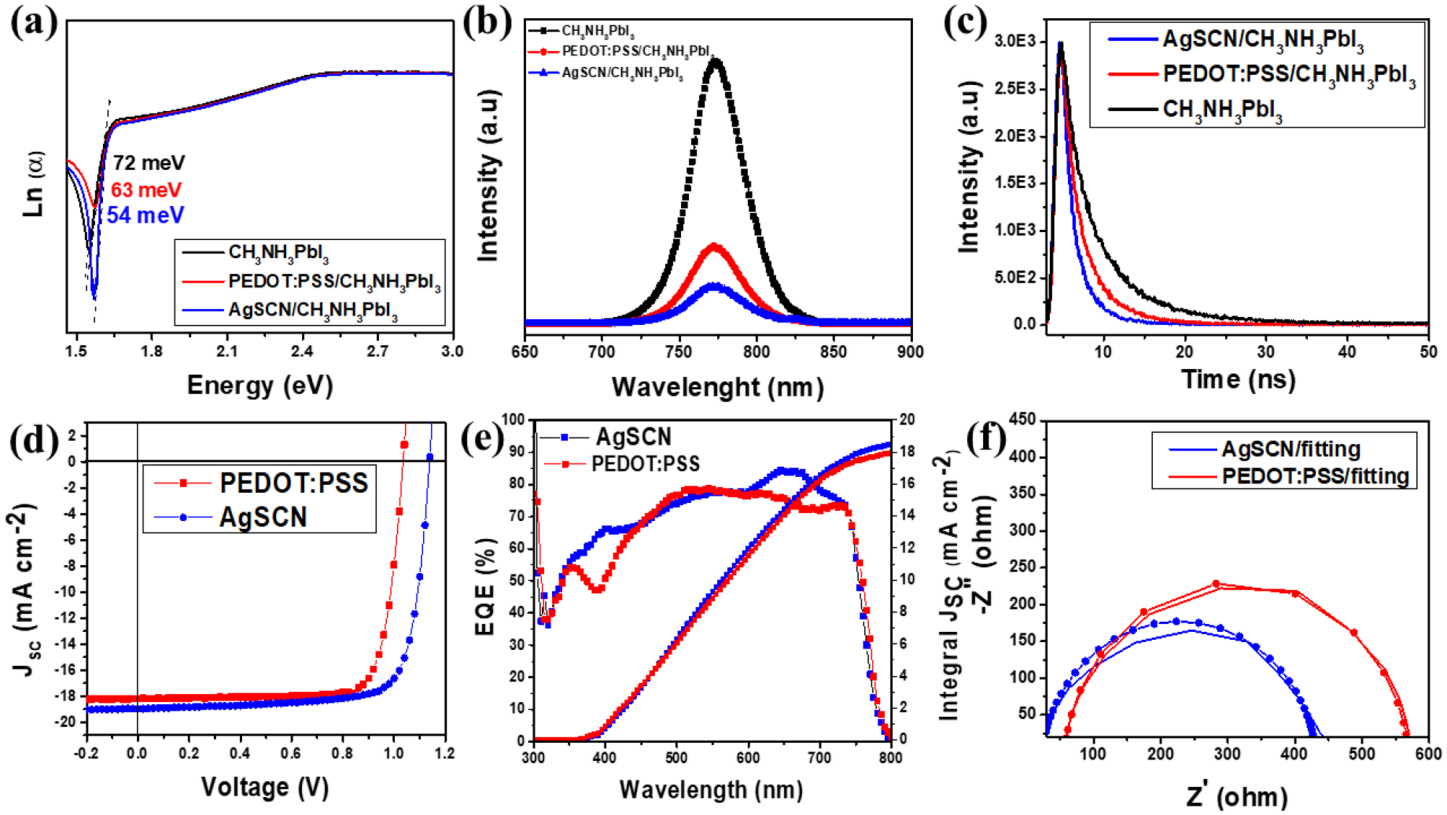
(**a**) Urbach energy (Eu) of CH_3_NH_3_PbI_3_ layers on substrates with and without AgSCN and PEDOT:PSS. (**b**) PL, (**c**) Using time-resolved photoluminescence (TRPL) spectroscopy, we compare the spectra of AgSCN and PEDOT:PSS thin films as HTL. (**d**) J − V curve for the prepared devices based on the optimum condition of AgSCN and PEDOT:PSS as a reference device. (**e**) Integrated J_SC_ and EQE spectra of the optimized AgSCN and PEDOT:PSS. (**f**) Devices made from PSCs and their corresponding Nyquist plot of AgSCN and PEDOT:PSS substrates at DC bias of 0.8 V under one sun AM1.5 illumination (the dotted lines give the fitted curves).

The absorbance of the active layer in perovskite solar cells is very significant in creating electron–hole pairs and consistently determines the final efficiency of the cells. Accordingly, the absorption spectra of ITO/AgSCN or PEDOT:PSS/CH_3_NH_3_PbI_3_ film and ITO/CH_3_NH_3_PbI_3_ film without HTM are compared in Fig. S4. The almost identical absorption behavior of the three films demonstrates that the choice of AgSCN as the HTL is better than PEDOT:PSS and does not affect the absorbing properties of CH_3_NH_3_PbI_3_ films. To study the effect of optoelectronic defects and crystallinity of the perovskite layer based on HTM of AgSCN or PEDOT:PSS. Urbach energy (E_u_) is derived from the absorption spectra (Fig. S4) based on the Urbach empirical rule described in SI details. Figure 5a shows the E_u_ of CH_3_NH_3_PbI_3_ layers on substrates with and without AgSCN and PEDOT:PSS. Urbach energy for AgSCN, PEDOT:PSS and CH_3_NH_3_PbI_3_ films have been calculated to be 54, 63, and 72 meV, respectively. It can be seen that the E_u_ value of CH_3_NH_3_PbI_3_ films without HTL is higher than AgSCN, PEDOT:PSS film which indicates more defect states and disordered atoms. Thus, the absorber layer improved based on AgSCN as HTM^40,41^.

Much work has been done to explore the beneficial features of AgSCN as HTL. To evaluate the charge extraction capabilities of the AgSCN-HTL layer, for instance, a steady-state photoluminescence (PL) quenching experiment has been set up. The hole extraction efficiency of PEDOT:PSS is compared to that of a reference AgSCN layer in Fig. 5b using CH_3_NH_3_PbI_3_ as the absorber. It is evident from Fig. 5b that AgSCN can extract holes more efficiently than PEDOT:PSS because the AgSCN film exhibits more efficient PL quenching.

In order to optimize the best concentration for preparing HTL film, we spin-coated the AgSCN with different concentrations (5, 15, 25, and 35 mg/mL). For the comparable study, PEDOT:PSS based a device is fabricated to ensure the conditions of cell fabrication. The optimized concentration of AgSCN was found to be 25 mg/mL. In addition, the measured hole mobility (${\upmu }_{h}$) for the 25 mg/ml of AgSCN was achieved $\sim$ 2.1 $\times$ 10^–2^ cm^2^/V/s compared to $\sim$ 5 $\times$ 10^–5^ of PEDOT:PSS. Figure S5 displays the measured current–voltage (JV) curves obtained in the reverse scan direction, and Table S2 summarizes the device's operational characteristics. Figure S5 and Table S2 show the highest PCE of 16.66% for AgSCN HTL fabricated from 25 mg/mL solution. For a value of V_OC_, 1.14 V, a FF of 77.01%, and a J_SC_ of 19.00 mA/cm^2^ are obtained for this champion device. The efficiency drops precipitously to 15.63% and 11.02% reductions in AgSCN concentration to 15 and 5 mg/mL, respectively. At the highest concentration of 35 mg/mL, the device achieved 14.33%, 1.11 V, 67.53%, and 19.17 mA/cm^2^ for PCE, V_OC_, FF, and J_SC_, respectively. As a result, the limit for AgSCN concentration should be set at no more than 25 mg/mL. Figure 5d compares the curves of J–V for the AgSCN- and PEDOT: PSS-HTL based best devices. The AgSCN-based PSC device presents a favorable performance, as compared to the 15.11% PCE of the PEDOT:PSS-HTL based device (V_OC_ = 1.04 V, J_SC_ = 18.17 mA/cm^2^, and FF = 0.80). The full parameters of V_OC_, J_SC_, FF and PCE are presented in Table 1. In AgSCN-based devices, higher film crystallinity and better charge transfer are compatible with SEM and PL characterizations, leading to increases in J_SC_ value and V_OC_.

**Table 1:** Device performance parameters of the perovskite solar cells with AgSCN and PEDOT:PSS as HTLs.

HTL	V_OC_ (V)	J_SC_ (mA/cm^2^)	FF	PCE (%)	R_s_ ($\Omega$)	R_sh_ ($\Omega$)	Integrated J_SC_^a^ (mA/cm^2^)
AgSCN (25 mg/mL)	1.14	19.00	77.01	16.66	16.22	653.12	18.87
PEDOT:PSS	1.04	18.17	80.37	15.11	22.45	550.41	18.01

^a^The integrated current density is calculated from EQE spectra.

Moreover, we have found that the AgSCN substrate has uniform surface morphology with small homogeneous particles ranging from 20 to 25 nm, and for the reference PEDOT:PSS in the same direction with homogeneous particles ranging from 20 to 30 nm, as shown in Fig. S6a,b. The successfully achieved thin film of AgSCN made a better perovskite film with free pinholes and large grains, while the thin film of PEDOT:PSS made a similar quality perovskite film with pinholes discovered, as presented in Fig. S6c,d. Moreover, the figures show that the perovskite layer's grain sizes are coordinated at 250–300 nm and 300–350 nm for PEDOT:PSS and AgSCN, respectively.

The J_SC_ of devices was also examined using EQE spectra or external quantum efficiency spectra (Fig. 5e). The integrated current density (J_SC_) from the EQE spectra was found to be 18.87 and 18.01 mA/cm^2^ for the AgSCN and PEDOT:PSS-HTL devices, respectively, which matches well with the J–V measurements. Also, electrochemical impedance spectroscopy (EIS) of the devices is conducted to test the effectiveness of AgSCN for charge transport at the PSC interfaces. Figure 5f displays the Nyquist plots for AgSCN and PEDOT: PSS devices as HTLs measured with AM1.5 illumination and a bias of 0.8 V, and their fitting findings for the corresponding curves are shown in the same Fig. 5f. The charge-transfer resistance (R_trans_) values of PEDOT:PSS and AgSCN based devices, respectively, were found to be 512.8 and 408.2 $\Omega$ from the fitting results (Table S3). The low R_trans_ value denotes the improved interfacial interaction between AgSCN and the CH_3_NH_3_PbI_3_ film, where the photo-generated holes are effectively extracted from the layer of perovskites. Accordingly, the system analysis using EIS analysis further verified that the AgSCN HTL would lead to improved charge transport and reduced charge recombination at device interfaces to provide enhanced photovoltaic efficiency of planar inverted PSCs.

Figure S7 (SI) and Table S4 compare the reproducibility of AgSCN and PEDOT:PSS HTLs within the PSC. The device statistics show parameters with limited distribution, including PCE of devices based on AgSCN HTL, indicating excellent reproducibility. In Fig. S8, the TGA and DTA were performed in an oxygen atmosphere between 30 and 600 °C to analyze the AgSCN's thermal stability. Figure S8 indicates an exothermic stage of AgSCN decay with a mass loss of 55% (Calcd. 55.80%) at 330 °C. Calculations of the mass loss revealed that AgO and Ag_2_O were the final products of decomposition. The superior thermal stability of the AgSCN at 550 °C can be accredited to a polymeric structure and the corresponding cyanide complex^42^.

On the other hand, improving PSC stability should be taken into account. As a result, our PSCs' stability under AM1.5 light and the temperature range (25–30 °C) has been tested for about 500 h without encapsulation. As shown in Fig. S9, the PSCs with AgSCN demonstrated exceptional long-term stability when compared to the control PEDOT:PSS based PSCs. The PCE of PSCs with AgSCN retains roughly 80.9 percent of its initial efficiency, which is altered from 16.7 to 13.52%, after operational measurement continuously for 500 h in an atmosphere with 48% relative humidity. While under the same testing conditions, the PCE of PEDOT:PSS based PSC is almost lowered from 15.11 to 9.43%, indicating lesser stability and a speedier rate of deterioration.

## Discussion

For inverted PSCs, silver thiocyanate (AgSCN) is a potential hole transporting material (HTM). Due to their superior reliability and high power conversion efficiency (PCE) compared to conventional perovskite solar cells, inverted PSCs have attracted much interest. However, finding an HTM appropriate for inverted PSCs can be difficult because the HTM must meet the energy levels of the perovskite and have high hole mobility, excellent stability, and compatibility. The high hole mobility of AgSCN (~ 2.1 × 10^–2^ cm^2^/V/s) has been shown to remove holes from the perovskite layer effectively. Energy loss at the perovskite-HTM contact is also mitigated by its good energy-level coordination with the perovskite. The higher work function for AgSCN than PEDOT:PSS (− 5.32 and − 5.1, respectively) matches the VB of the CH_3_NH_3_PbI_3_ as active layer than PEDOT:PSS, which reduces the energy loss for h^+^ transfer from the VB of CH_3_NH_3_PbI_3_ to AgSCN. Therefore, the enhanced work function may also boost the V_OC_ of the device by increasing the possible distinction between the HTL VB and the perovskite layer^18^. This can result in a higher V_OC_ for the PSC, as there is a more significant driving force for the transfer of charge carriers from the perovskite layer to the HTL.

AgSCN's high temperature stability also bodes well for the IPSC device's durability. As can be shown in Fig. 5c, the results of the time-resolved PL (TRPL) are consistent with those of the steady-state PL quenching. These results highlight the need of effective charge extraction and collection by demonstrating the existence of an efficient hole transfer between perovskite and AgSCN. The EQE value of the somewhat higher AgSCN-HTL based device is attributed to improved charge extraction and enhanced charge collection, which indicates better device performance. The improved open-circuit voltage (V_OC_) in the AgSCN-HTL based device suggests less recombination, which is consistent with the idea that AgSCN-HTL reduces trap states and improves energy band alignment. Its concludes that the first-round try of the perovskite solar cell (PSC) efficiency using AgSCN-HTL is significantly superior than that of most devices using PEDOT:PSS-HTL, and further improvement can be accomplished by optimizing the perovskite layer and/or electron transport layer (ETL) thicknesses. Overall, this paragraph provides evidence that the use of AgSCN-HTL results in improved performance of PSCs compared to PEDOT:PSS-HTL, by reducing trap states, improving energy band alignment, and enhancing charge collection. This is important because it suggests that optimizing the choice of HTL can improve the overall efficiency of PSCs, which is crucial for their widespread adoption as a sustainable energy source.

Regarding the inferior stability of PEDOT:PSS-based devices, the effect of PEDOT:PSS on the stability of PSCs has been extensively studied, and based on the stability test in this work, there is evidence to suggest that the acidic nature of PEDOT:PSS can cause degradation over time. It has been shown that PEDOT:PSS can release protons and acidify the perovskite layer, leading to the formation of lead iodide and the loss of performance in the solar cell^43–45^. While for AgSCN, the dipropyl sulfide used as a solvent is very volatile, and within the spin coating, it does not affect the degradation of the perovskite layer.

In summary, AgSCN is a promising HTM for inverted PSCs due to its high hole mobility, appropriate energy level alignment, and good stability. Further studies are needed to optimize its performance and understand the underlying mechanisms, but it has the potential to enable the development of more efficient and stable IPSC devices.

## Conclusion

In conclusion, we used for the first time AgSCN as HTL in a room-temperature solution process to create CH_3_NH_3_PbI_3_-based PSC, and its insertion into planar p-i-n configuration PSC increased efficiency by 16.66%. When AgSCN is used as the HTL, solar cells have PCE more than 1% higher than when PEDOT:PSS is utilized as the HTL. This difference is primarily attributable to a dramatic rise in the V_OC_ of 0.1 V and a slight increase in FF and J_SC_. The experimental data revealed superior results, primarily due to favorable energy level distribution, excellent mobility of charge, and ability to extract holes. The hole mobility for the 25 mg/ml of AgSCN was achieved $\sim$ 2.1 $\times$ 10^–2^ cm^2^/V/s compared to $\sim$ 5 $\times$ 10^–5^. This low-temperature, low-cost AgSCN production method is seen as easy and scalable, which bodes well for commercializing perovskite-based flexible devices and photovoltaic technologies. In sum, AgSCN is a non-toxic, inexpensive rival that is simple to work with at low temperatures. It is a strong contender for creating tandem devices and high-efficiency p-i-n junction solar cells made of perovskites.

## Supplementary Information

Supplementary Information.

## Data Availability

All data generated or analyzed during this study are included in this published article and will be available on request through the corresponding author.
