# Supplementary material for: AgSCN as a new hole transporting material for inverted perovskite solar cells
Source: Sci Rep. 2023 May 16;13:7939. doi: 10.1038/s41598-023-35081-z (PMC10188483; doi:10.1038/s41598-023-35081-z)
Supplement: Supplementary file 1 — Supplementary Information. [file 41598_2023_35081_MOESM1_ESM.docx]

**Supplementary file**

**AgSCN as a new hole transporting material for inverted perovskite solar cells**

*Ahmed Mourtada Elseman*

Electronic & Magnetic Materials Department, Advanced Materials Division, Central Metallurgical Research and Development Institute (CMRDI), Helwan, P.O. Box 87, Cairo 11421, Egypt.

Email: amourtada@cmrdi.sci.eg

**Materials**

PEDOT:PSS (4083), methylammonium iodide (MAI, 99.5%), PbI_2_ (99.99%), PC_61_BM (99%), BCP (99%) were purchased form Xi'an Polymer Light Technology Corp (China), while N, N-dimethylformide (DMF, 99.8%), dimethyl sulfoxide (DMSO, 99.9%) and chlorobenzene (CB, 99.8%) were purchased from Sigma-Aldrich.

Silver chloride (AgCl) and ammonium thiocyanate (NH_4_SCN) are starting materials for synthesizing AgSCN were purchased from Alfa Aesar. PEDOT:PSS (P VP AI 4083) from Sigma-Aldrich was used as reference HTL. The AgSCN and PEDOT:PSS were filtered through a sterile syringe filter PTFE 0.45 $\mu$m before fabrication. The perovskite layer was synthesized through hybrid solutions (1.5 M) of PbI_2_ (461 mg), and MAI (159 mg) was attained in a DMF and DMSO co-solvent (vol. ratio = 600:78) for 12 hours with stirring at room temperature in a glove box overnight and then filtered by PTFE 0.22 $\mu$m before use. Overnight, combine 20 mg/1 ml CB PC_61_BM and BCP 0.5 mg/1 mL ethanol at room temperature.

**UPS Calculations**

Work function and electron affinity are among the most critical properties of semiconductors, which play essential roles in functional properties and device performance once interfaces or junctions are involved, for example, metal-semiconductor junctions in devices, or hetero-catalytic materials, hetero- or homo-junctions for photovoltaic cells, photonic devices, and composites as environmental catalysts. The work function, Φ, is the energy needed to remove an electron from the surface of a condensed solid into the external vacuum[^1-5^](#_ENREF_1).

The work function and valence band maximum (VBM) of AgSCN were measured by UPS to be –5.07 ± 0.1 eV. The work function was calculated by subtracting the He I radiation energy of 21.2 eV from the high-binding energy cutoff at 16.15 eV (Fig. 4b): 16.15 (± 0.1) eV – 21.2 eV = –5.07 ± 0.1 eV versus vacuum. Similarly, for a bare AgSCN surface, the low-energy tail of the UPS spectrum was used to determine the position of the VBM as 0.25 ± 0.1 eV below the Fermi level. Therefore, the VBM was calculated to be –5.07 (± 0.1) eV – 0.25 (± 0.1) eV = –5.32 ± 0.2 eV versus vacuum.

**Calculation of Optical Bang gap**

The optical band gap was estimated using Tauc’s equations:

*αhʋ = A(hy ^_^ E_g_)^n^* (1)

where n equals 1/2 and 2 for direct and indirect transitions, respectively, and α is the absorption coefficient. A is an energy-independent constant. The plots of *(αhʋ)^2^* and *(αhʋ)^1/2^* versus hʋ have been produced, from which a direct band gap was found in Fig. 4c by extrapolating the linear portion of the curve to

*(αhʋ)^1/2^* = 0 (2)

The curves indicate that the direct band gap Eg value equals 3.95 ± 0.1 *eV* for AgSCN. Considering the optical bandgap of ~3.95 eV, the conduction band minimum (CBM) was calculated from equation (3).

*Ec = Ev – Eg* (3)

(*Ec = -5.32* ± 0.2 *– 3.95*± 0.1) to be –1.37± 0.3 eV versus vacuum.

**Calculation of Urbach Energy (E_u_)**

UV–Visible spectra of the ITO/AgSCN or PEDOT:PSS/CH_3_NH_3_PbI_3_ film and ITO/CH_3_NH_3_PbI_3_ film without HTM have not shown much difference in the absorption nature of the films, as shown in **Fig. S4**. The Urbach energy (E_u_) is the parameter that can be related to the material's crystallinity, defects, and optoelectronic characteristics. Near the optical band edge, the connection between absorbance coefficient ($\alpha$) and energy (*hv*) can be explained as the Urbach empirical rule. The E_u_ is obtained from the slope near the optical band-edge[^6^](#_ENREF_6).

$\alpha= \alpha_{0}\exp\left( \frac{hv}{E_{u}} \right)$ (4)

$\ln(\alpha)= {\ln(\alpha}_{0})+ \left( \frac{hv}{E_{u}} \right)$ (5)

Here, *hv* = energy of incident photon and E_u_ = Urbach energy of the defect states in the bandgap, and $\alpha_{0}$ = constant (intercept). Therefore, comparing the above equation with the simple equation, *y = c + mx*, where *m* is the slope.

$E_{u}= \frac{1}{slope}$ (6)


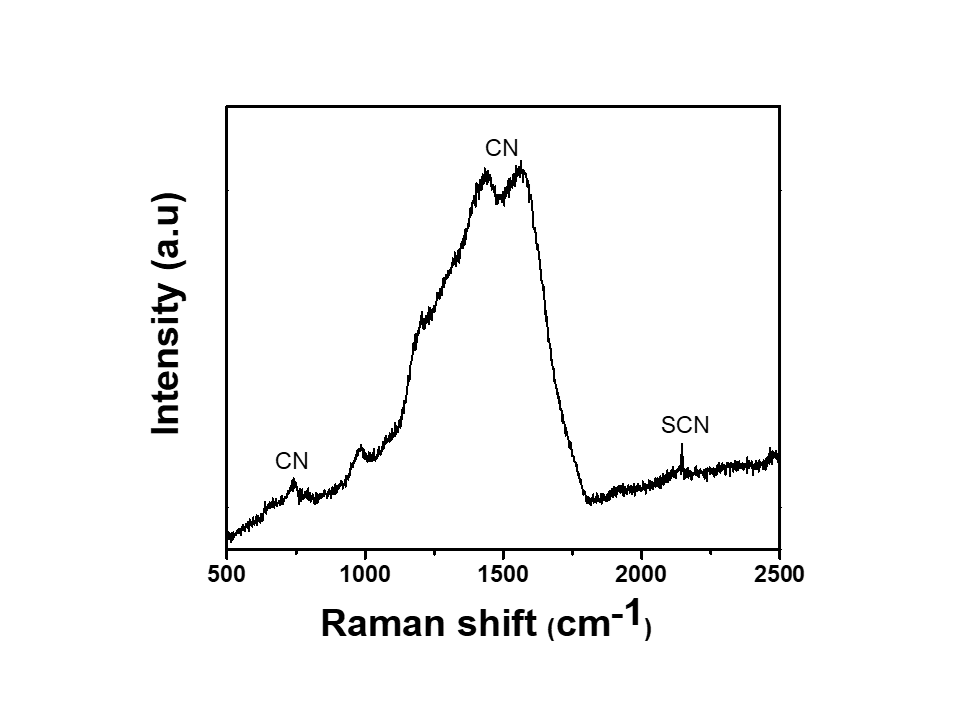


**Fig. S1.** Raman spectroscopy analysis of the invented hole transport material, AgSCN.


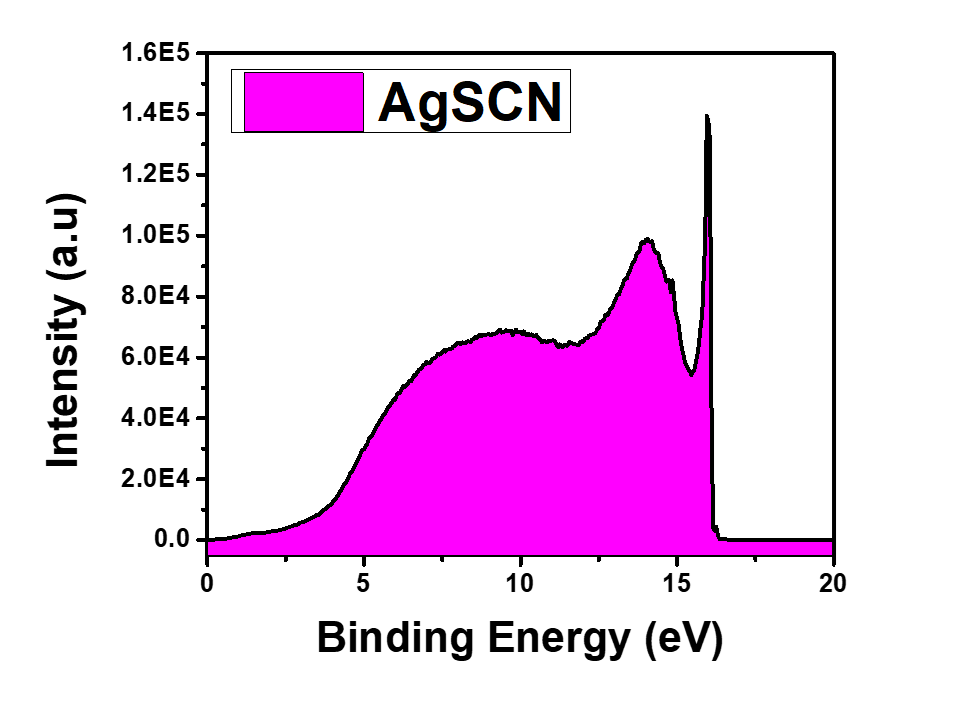


**Fig. S2.** (a) UPS spectrum of AgSCN.


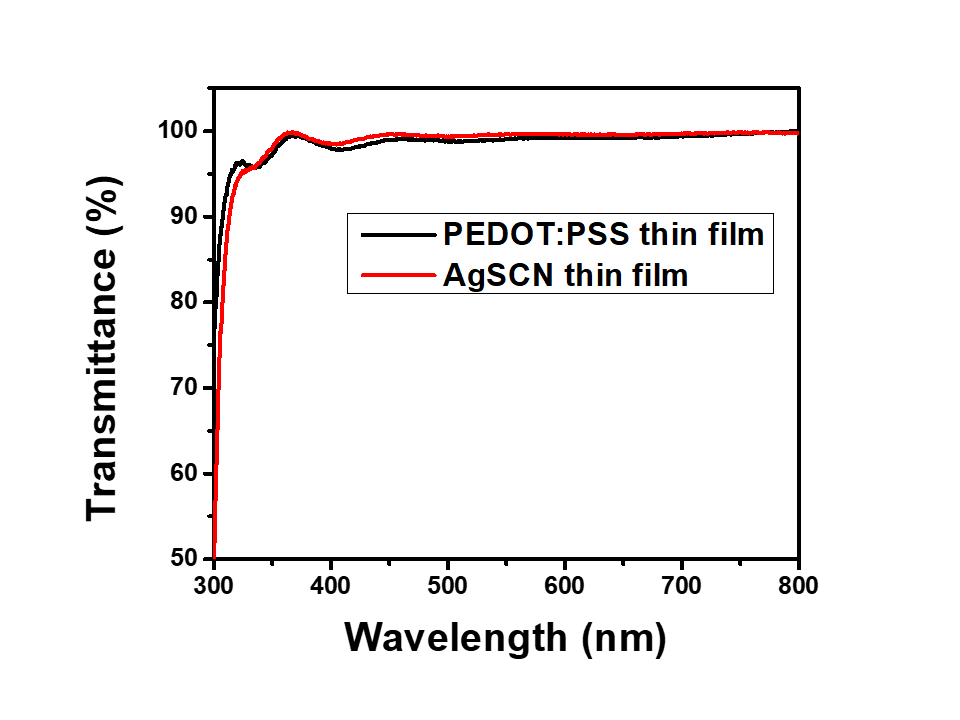


**Fig. S3.** (a) Transmittance spectra of AgSCN and PEDOT:PSS on ITO substrates.


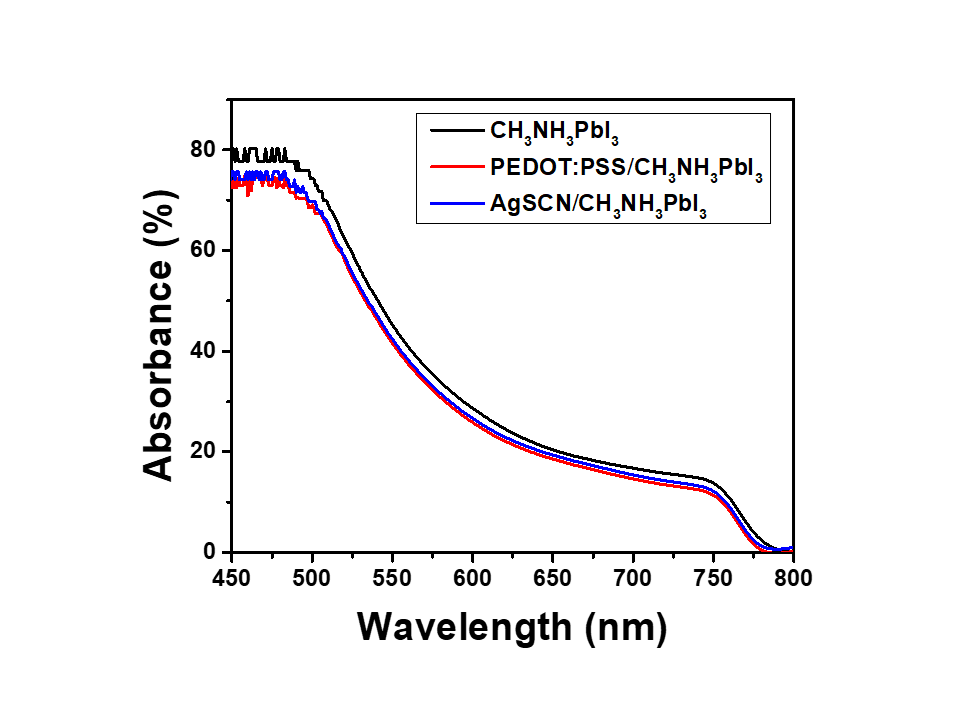


**Fig. S4**. (a) Absorption spectra of CH_3_NH_3_PbI_3_ layers on substrates with and without AgSCN and PEDOT:PSS.


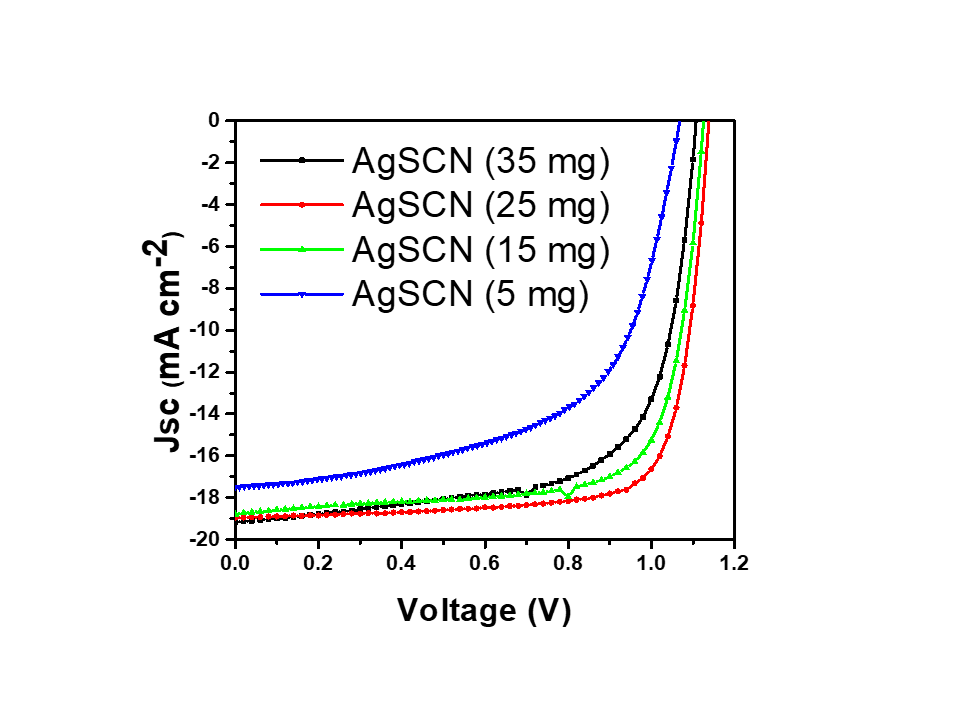


**Fig. S5**. J−V characteristics for the optimum devices based on AgSCN with different concentrations.


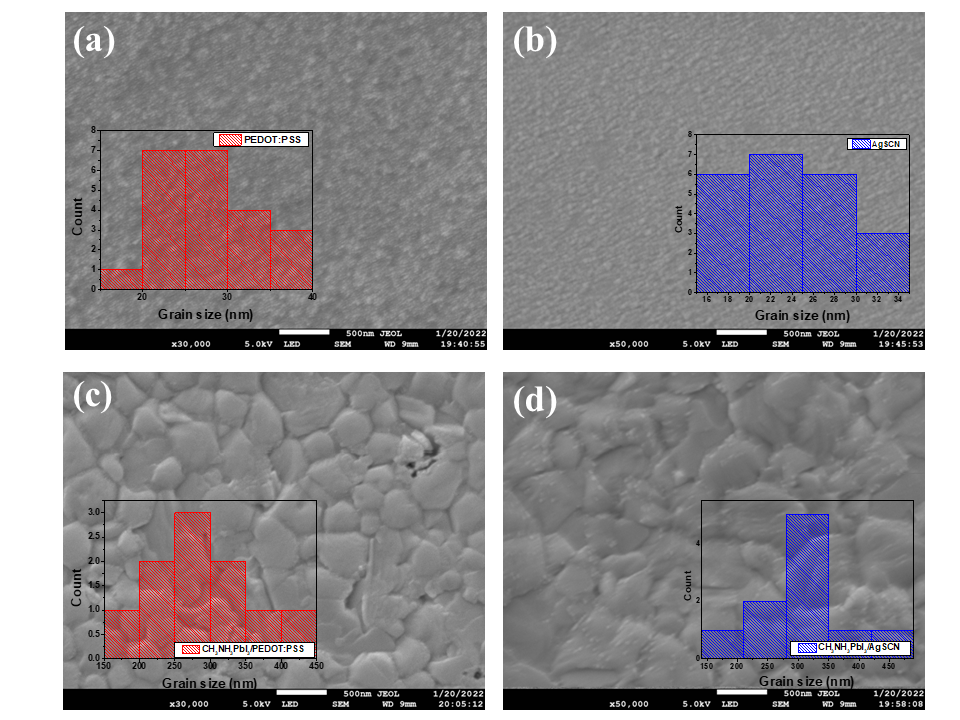


**Fig. S6.** SEM top-view images of (a) PEDOT:PSS substrate, (b) AgSCN substrate, (c) and (d) perovskite film on the PEDOT:PSS and AgSCN substrate, respectively. Inside grain size distribution for each, respectively.


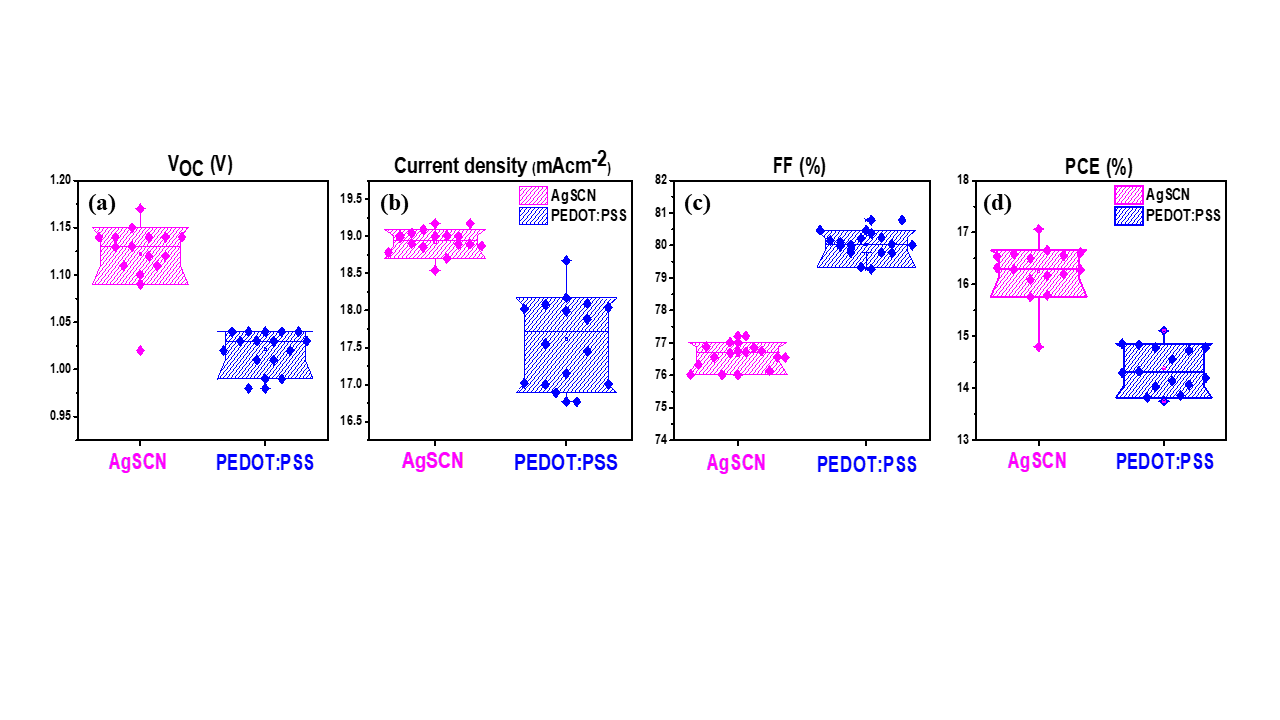


**Fig. S7.** The box chart of V_OC_, J_SC_, FF, and PCE of perovskite solar cells fabricated on AgSCN and PEDOT:PSS substrates. The chart is drawn from 16 devices for each HTL.


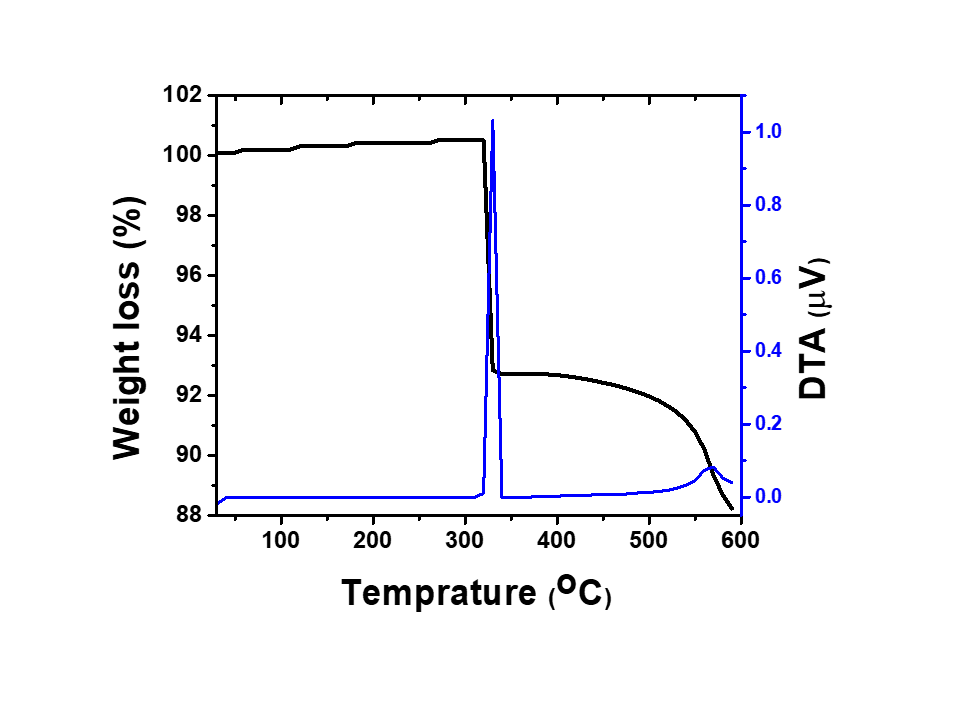


**Fig. S8.** Thermogravimetric (TGA) and differential thermal analyses (DTA) to examine the thermal stability of the AgSCN.


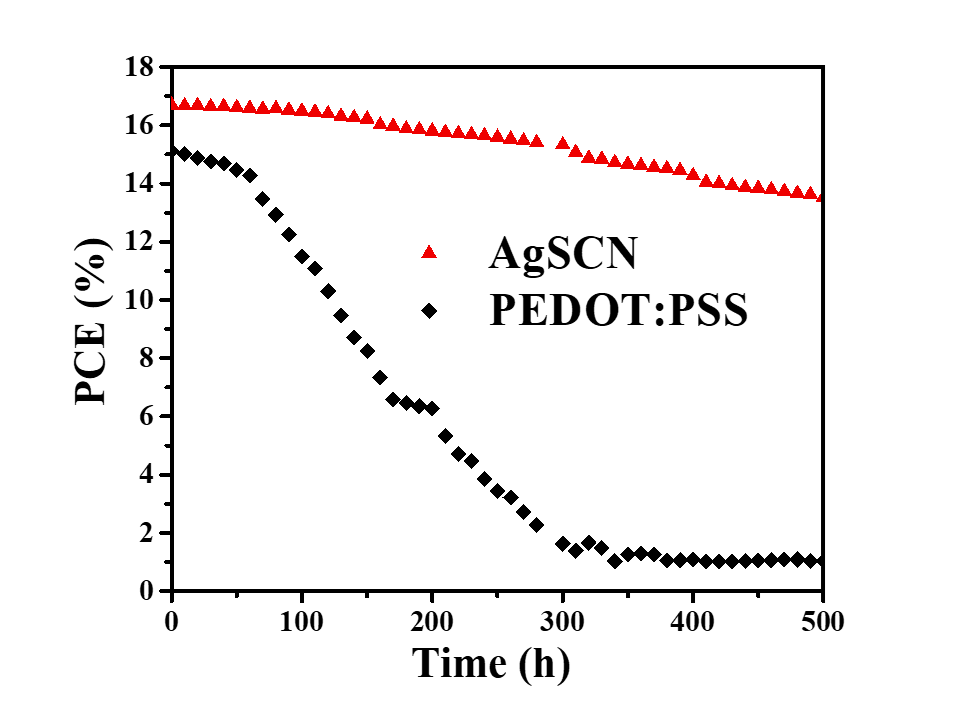


**Fig. S9.** The stability of perovskite solar cells under an ambient environment of 48% relatively humidity without encapsulation at room temperature to examine the stability of the AgSCN as HTL.

**Table S1.** Photovoltaic Parameters of PSCs Based on CuSCN Inorganic HTM.

| Deposition method of CuSCN | HTM structure | Device structure | Perovskite structure | J_SC_ (mA/cm^2^ ) | V_OC_ (V) | FF | PCE (%) | year | ref |
| --- | --- | --- | --- | --- | --- | --- | --- | --- | --- |
| spin-coating | CuSCN | inverted | MAPbI_3_ | 15.70 | 1.06 | 0.65 | 10.80 | 2015 | [^7^](#_ENREF_7) |
| electrodeposition | CuSCN | inverted | MAPbI_3_ | 21.90 | 1.00 | 0.76 | 15.60 | 2015 | [^8^](#_ENREF_8) |
| spin-coating | CuSCN/NH_3_ | inverted | MAPbI_3_ | 22.70 | 1.10 | 0.71 | 17.50 | 2017 | [^9^](#_ENREF_9) |
| electrodeposition | CuSCN | inverted | MAPbI_3_ | 19.79 | 0.90 | 0.64 | 11.40 | 2017 | [^10^](#_ENREF_10) |
| spin-coating | CuSCN/r-GO/ | inverted | MAPbI_3_ | 18.21 | 1.03 | 0.76 | 14.28 | 2018 | [^11^](#_ENREF_11) |
| spin-coating | CuSCN/F4TCNQ | inverted | MAPbI_3_ | 21.01 | 0.99 | 0.72 | 15.01 | 2019 | [^12^](#_ENREF_12) |
| spin-coating | CuSCN/PEDOT:PSS | inverted | MAPbI_3_ | 19.10 | 1.0 | 78.5 | 15.30 | 2020 | [^13^](#_ENREF_13) |

**Table S2.** Device performance parameters of the perovskite solar cells with diﬀerent concentrations of AgSCN as hole transport layer.

| HTL (mg/mL) | V_OC_ (V) | J_SC_ (mA/cm^2^) | FF | PCE (%) |
| --- | --- | --- | --- | --- |
| AgSCN (5) | 1.07 | 17.50 | 58.98 | 11.02 |
| AgSCN (15) | 1.13 | 18.83 | 73.73 | 15.63 |
| AgSCN (25) | 1.14 | 19.00 | 77.01 | 16.66 |
| AgSCN (35) | 1.11 | 19.17 | 67.53 | 14.33 |

**Table S3.** Fitting data for Nyquist plots with the equivalent circuit by Zview.

| HTL | R_rec_(Ω) | R_trans_(Ω) |
| --- | --- | --- |
| PEDOT:PSS | 56.39 | 512.8 |
| AgSCN (25 mg/mL) | 23.19 | 408.2 |

**Table S4.** Photovoltaic parameters of thirty PSCs devices made of AgSCN under simulated AM-1.5G illumination (power density 100 mW/cm^2^).

| **No. of cells** | **V_OC_ (V)** | **J_SC_(mA/cm^2^)** | **FF (%)** | **PCE (%)** |
| --- | --- | --- | --- | --- |
| 1 | 1.14 | 19.00 | 77.01 | 16.66 |
| 2 | 1.10 | 19.09 | 77.02 | 16.17 |
| 3 | 1.11 | 18.54 | 76.75 | 15.79 |
| 4 | 1.09 | 18.85 | 76.69 | 15.76 |
| 5 | 1.15 | 18.70 | 76.72 | 16.50 |
| 6 | 1.12 | 18.90 | 76.01 | 16.09 |
| 7 | 1.14 | 18.89 | 76.86 | 16.55 |
| 8 | 1.14 | 19.01 | 76.56 | 16.59 |
| 9 | 1.14 | 18.98 | 76.75 | 16.61 |
| 10 | 1.13 | 19.04 | 76.88 | 16.54 |
| 11 | 1.17 | 18.89 | 77.21 | 17.06 |
| 12 | 1.12 | 19.00 | 76.14 | 16.20 |
| 13 | 1.02 | 19.01 | 76.33 | 14.80 |
| 14 | 1.11 | 19.17 | 76.57 | 16.29 |
| 15 | 1.14 | 18.78 | 76.02 | 16.28 |
| 16 | 1.13 | 18.87 | 76.55 | 16.32 |
| **Average** | 1.099 $\pm$ 0.02 | 18.80 $\pm$ 0.12 | 76.35 $\pm$ 0.28 | 15.92 $\pm$ 0.35 |

**Table S5.** Photovoltaic parameters of thirty PSCs devices made of PEDOT:PSS under simulated AM-1.5G illumination (power density 100 mW/cm^2^).

| **No. of cells** | **V_OC_ (V)** | **J_SC_(mA/cm^2^)** | **FF (%)** | **PCE (%)** |
| --- | --- | --- | --- | --- |
| 1 | 1.04 | 18.17 | 80.37 | 15.11 |
| 2 | 1.04 | 17.15 | 79.27 | 14.14 |
| 3 | 1.04 | 17.00 | 79.33 | 14.03 |
| 4 | 1.04 | 17.45 | 80.22 | 14.56 |
| 5 | 1.03 | 17.02 | 80.24 | 14.07 |
| 6 | 1.01 | 17.01 | 80.02 | 13.75 |
| 7 | 1.03 | 17.99 | 79.78 | 14.78 |
| 8 | 1.02 | 18.08 | 79.89 | 14.73 |
| 9 | 0.99 | 18.09 | 80.04 | 14.33 |
| 10 | 1.01 | 17.55 | 80.10 | 14.20 |
| 11 | 1.04 | 17.88 | 79.78 | 14.84 |
| 12 | 0.98 | 18.67 | 80.78 | 14.78 |
| 13 | 0.99 | 18.02 | 80.15 | 14.30 |
| 14 | 1.03 | 16.77 | 80.01 | 13.82 |
| 15 | 1.03 | 18.04 | 79.98 | 14.86 |
| 16 | 1.02 | 16.89 | 80.47 | 13.86 |
| **Average** | 1.01 $\pm$ 0.02 | 17.11 $\pm$ 0.51 | 79.76 $\pm$ 0.27 | 14.01 $\pm$ 0.38 |

**References**

1 Greczynski, G. & Hultman, L. X-ray photoelectron spectroscopy: Towards reliable binding energy referencing. *Progress in Materials Science* **107**, 100591, (2020).

2 Elseman, A. M., Luo, L. & Song, Q. L. Self-doping synthesis of trivalent Ni_2_O_3_ as a hole transport layer for high fill factor and efficient inverted perovskite solar cells. *Dalton Transactions* **49**, 14243-14250 (2020).

3 Elseman, A. M. *et al.* Efficient and Stable Planar n-i-p Perovskite Solar Cells with Negligible Hysteresis through Solution-Processed Cu_2_O Nanocubes as a Low-Cost Hole-Transport Material. *ChemSusChem* **12**, 3808-3816 (2019).

4 Fabian, D. M. & Ardo, S. Hybrid organic–inorganic solar cells based on bismuth iodide and 1,6-hexanediammonium dication. *Journal of Materials Chemistry A* **4**, 6837-6841 (2016).

5 Shao, G. Work Function and Electron Affinity of Semiconductors: Doping Effect and Complication due to Fermi Level Pinning. *Energy & Environmental Materials* **4**, 273-276 (2021).

6 Wu, S. *et al.* Efficient large guanidinium mixed perovskite solar cells with enhanced photovoltage and low energy losses. *Chemical Communications* **55**, 4315-4318 (2019).

7 Zhao, K. *et al.* Solution-processed inorganic copper (I) thiocyanate (CuSCN) hole transporting layers for efficient p–i–n perovskite solar cells. *Journal of Materials Chemistry A* **3**, 20554-20559 (2015).

8 Ye, S. *et al.* CuSCN-based inverted planar perovskite solar cell with an average PCE of 15.6%. *Nano Lett.* **15**, 3723-3728 (2015).

9 Wijeyasinghe, N. *et al.* Copper (I) thiocyanate (CuSCN) hole‐transport layers processed from aqueous precursor solutions and their application in thin‐film transistors and highly efficient organic and organometal halide perovskite solar cells. *Adv. Funct. Mater.* **27**, 1701818 (2017).

10 Xi, Q. *et al.* Highly efficient inverted solar cells based on perovskite grown nanostructures mediated by CuSCN. *Nanoscale* **9**, 6136-6144 (2017).

11 Chowdhury, T. H. *et al.* Low temperature processed inverted planar perovskite solar cells by r-GO/CuSCN hole-transport bilayer with improved stability. *Solar Energy* **171**, 652-657 (2018).

12 Jin, I. S., Lee, J. H., Noh, Y. W., Park, S. H. & Jung, J. W. Molecular doping of CuSCN for hole transporting layers in inverted-type planar perovskite solar cells. *Inorganic Chemistry Frontiers* **6**, 2158-2166 (2019).

13 Xu, L. *et al.* Improving the efficiency and stability of inverted perovskite solar cells by CuSCN-doped PEDOT:PSS. *Sol. Energy Mater. Sol. Cells* **206**, 110316 (2020).
